# Acute Promyelocytic Leukemia with double minute chromosomes: a rare case with high relapse risk

**DOI:** 10.1093/omcr/omaf259

**Published:** 2025-12-26

**Authors:** Yohei Sasaki, Shotaro Shimada, Natsuki Kawamata, Hidenori Hayashi, Kazuki Nagao, Kai Kuroiwa, Hinako Narita, Reiko Okamura, Megumi Watanuki, Nana Arai, Kouji Yanagisawa, Norimichi Hattori

**Affiliations:** Division of Hematology, Department of Medicine, Showa Medical University School of Medicine, 1-5-8 Hatanodai, Shinagawa-Ku, Tokyo 142-8666, Japan; Division of Hematology, Department of Medicine, Showa Medical University School of Medicine, 1-5-8 Hatanodai, Shinagawa-Ku, Tokyo 142-8666, Japan; Division of Hematology, Department of Medicine, Showa Medical University School of Medicine, 1-5-8 Hatanodai, Shinagawa-Ku, Tokyo 142-8666, Japan; Division of Hematology, Department of Medicine, Showa Medical University School of Medicine, 1-5-8 Hatanodai, Shinagawa-Ku, Tokyo 142-8666, Japan; Division of Hematology, Department of Medicine, Showa Medical University School of Medicine, 1-5-8 Hatanodai, Shinagawa-Ku, Tokyo 142-8666, Japan; Division of Hematology, Department of Medicine, Showa Medical University School of Medicine, 1-5-8 Hatanodai, Shinagawa-Ku, Tokyo 142-8666, Japan; Division of Hematology, Department of Medicine, Showa Medical University School of Medicine, 1-5-8 Hatanodai, Shinagawa-Ku, Tokyo 142-8666, Japan; Division of Hematology, Department of Medicine, Showa Medical University School of Medicine, 1-5-8 Hatanodai, Shinagawa-Ku, Tokyo 142-8666, Japan; Division of Hematology, Department of Medicine, Showa Medical University School of Medicine, 1-5-8 Hatanodai, Shinagawa-Ku, Tokyo 142-8666, Japan; Division of Hematology, Department of Medicine, Showa Medical University School of Medicine, 1-5-8 Hatanodai, Shinagawa-Ku, Tokyo 142-8666, Japan; Division of Hematology, Department of Medicine, Showa Medical University School of Medicine, 1-5-8 Hatanodai, Shinagawa-Ku, Tokyo 142-8666, Japan; Division of Hematology, Department of Medicine, Showa Medical University School of Medicine, 1-5-8 Hatanodai, Shinagawa-Ku, Tokyo 142-8666, Japan

**Keywords:** acute promyelocytic leukemia, double minutes chromosomes, differentiation therapy, relapse

## Abstract

Acute promyelocytic leukemia (APL) is a subtype of acute myeloid leukemia (AML); it has a high response rate and long-term survival with differentiation therapy and chemotherapy. However, only one previous case of APL with double minute chromosomes (DMs, a poor prognostic factor for AML) has been reported. We report the case of a patient with APL and DMs. A 44-year-old woman was treated with all-trans retinoic acid (ATRA) and chemotherapy and achieved molecular complete remission (mCR). However, the condition relapsed after 15 months. She was treated with arsenic trioxide and autologous transplantation and experienced mCR. Her peripheral blood was positive for minimal residual disease (MRD) 2 months after autologous transplantation. She became MRD-negative with ATRA and has maintained the negative status for 15 months. This is the first report to suggest that patients with APL and DMs may be a high-risk group for relapse and benefit from maintaining with ATRA.

## Introduction

Acute promyelocytic leukemia (APL) is a subtype of acute myeloid leukemia (AML). AML exhibits characteristic findings on cross-sectional imaging, and fusion modalities such as 2-deoxy-2-[18F]fluoro-d-glucose positron emission tomography/computed tomography can assess various AML subtypes [1, 2]. However, bone marrow examination and genetic examination are required for definitive diagnosis and classification of AML. Almost all cases of APL exhibit a *PML::RARA* genetic rearrangement resulting from the chromosomal translocation t(15;17)(q24;q21) and are highly responsive to differentiation therapy with all-trans retinoic acid (ATRA) or arsenic trioxide (ATO). Treatment with ATRA and ATO is the preferred first-line treatment for standard-risk APL [3]. On the other hand, treatment with ATRA and chemotherapy has achieved long-term survival rates exceeding 80% [4]. Thus, ATRA and chemotherapy remain a viable alternative treatment, particularly for high-risk cases or countries where ATRA and ATO are not adapted for first-line treatment. However, relapse occurs in approximately 20% of these patients. Therefore, induction and consolidation therapy with ATO, high-dose cytarabine chemotherapy, and autologous hematopoietic cell transplantation are required for patients with relapsed APL [5]. White blood cell (WBC) and platelet counts and gene mutations have been identified as high-risk factors for relapse in patients with APL [6–8]. However, the impact of additional chromosomal abnormalities on t(15;17) is controversial [9].

Double minute chromosomes (DMs) have been linked to aggressive disease and poor prognosis in patients with AML [10]. However, the clinical relevance of DMs in patients with APL remains uncertain due to the limited reports. Herein, we report the case of a patient with relapsed APL, with DMs.

## Case report

**Figure 1:**
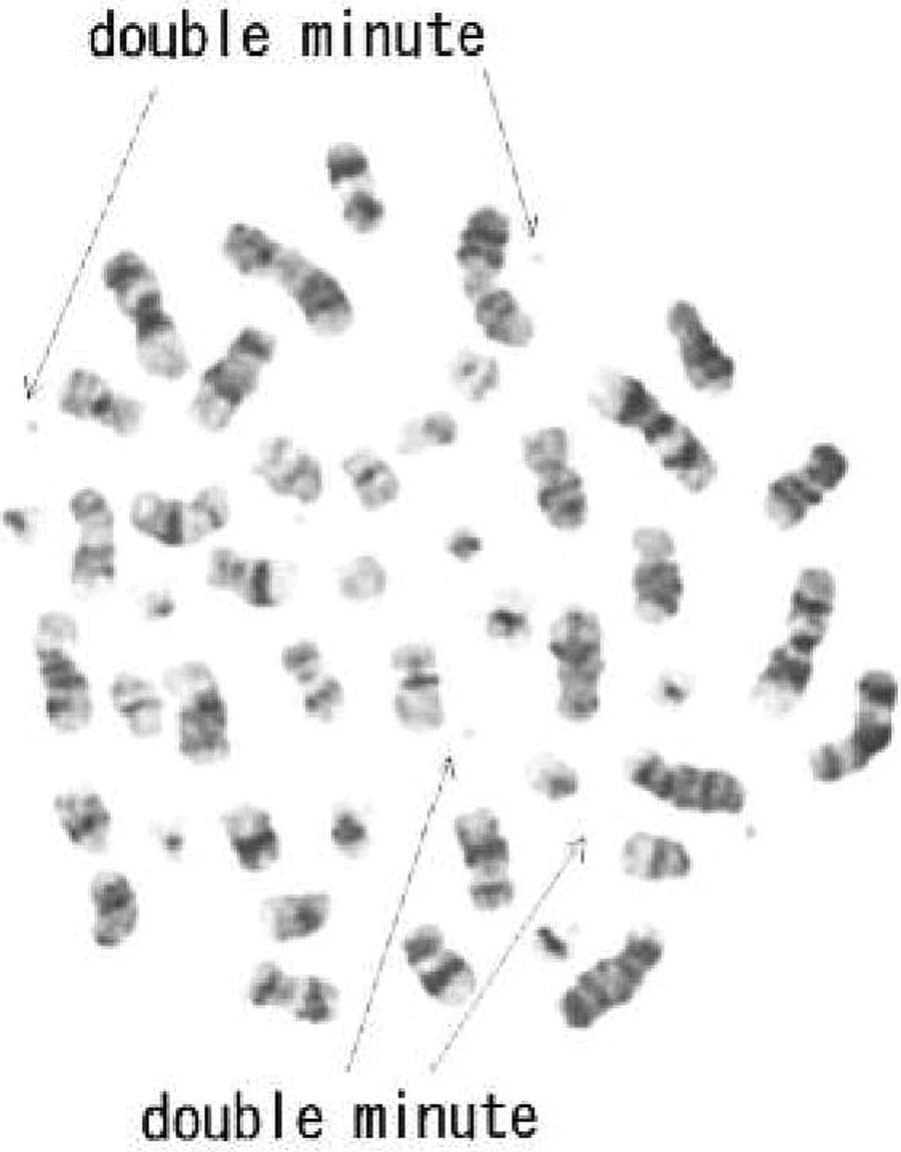
The bone marrow chromosomes show that 15 of the 20 cells had 46, XX, del (9)(q22q34), t(15:17)(q24;q21), and 13 of those 15 cells had 1–21 double minute chromosomes.

**Figure 2:**
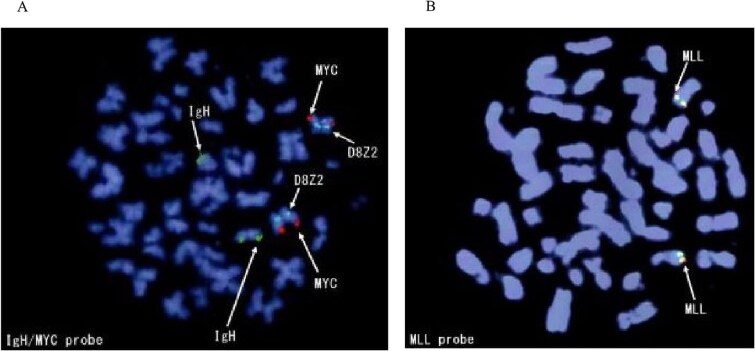
Fluorescence in-situ hybridization showed that MYC and MLL rearrangements were not detected.

**Figure 3:**
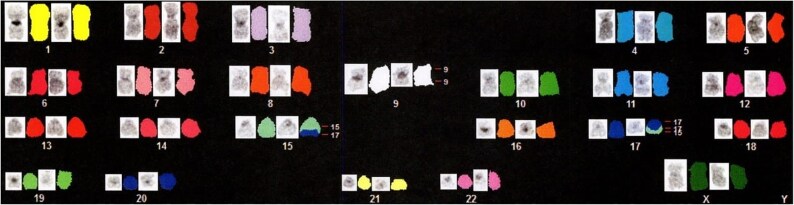
Spectral karyotyping shows a deletion of chromosome 9 and translocation of chromosomes 15 and 17, but cannot detect the chromosome of origin of the double minute chromosomes.

A 44-year-old woman was admitted to our hospital for pancytopenia in March 2022. She had a 2-week history of fever, and her past medical history included mastitis, appendicitis, and surgically treated thyroid follicular carcinoma. Her WBC count was 3100/μL, with a promyelocyte ratio of 33%, a hemoglobin level of 7.2 g/dL, and a platelet count of 0.4 × 10^9^/μL. The smear of bone marrow (BM) aspirates revealed a nucleated cell count of 258 000/μL, with 87.2% promyelocyte cells exhibiting irregular nuclear shapes, azure granules, and basophilic cytoplasmic margins, as well as cytoplasmic acidophilic features. Flow cytometry and immunohistochemistry of the BM biopsy specimens revealed the following immunophenotypes: cluster of differentiation (CD)13 (+), CD33 (+), CD34 (−), CD56 (−), CD117 (−), human leukocyte antigen-DR (−), terminal deoxynucleotidyl transferase (−), and myeloperoxidase (+). Karyotype analysis revealed that 15 of the 20 cells had 46, XX, del [9](q22q34), t(15:17)(q24;q21), and 13 of those 15 cells had 1–21 DMs (Fig. 1). Fluorescence in-situ hybridization revealed that *PML:RARA* translocation was positive, whereas *MYC* and *MLL* rearrangements were not detected (Fig. 2). Spectral karyotyping failed to detect the chromosome of origin of the DMs (Fig. 3). Real-time polymerase chain reaction (PCR) revealed *PML::RARA*. The patient was diagnosed with APL. PCR also revealed no *FLT3-ITD*, *NPM1*, *IDH1/2*, *SF3B1*, *ASXL1*, and *DNMT3A* mutations.

Because ATO is not approved for use in patients with primary APL under the Japanese National Health Insurance, ATRA and ATO were not adapted for first-line treatment. Therefore, the patient was treated with ATRA and induction chemotherapy with cytarabine (50 mg/m^2^ × 5 days) and idarubicin (12 mg/m^2^ × 2 days) in March 2022. On day 27, the BM aspirate revealed hematological complete remission (CR). She received the first cycle of consolidation chemotherapy with cytarabine (100 mg/m^2^ × 5 days) and mitoxantrone (7 mg/m^2^ × 3 days). On day 23, the BM aspirate revealed molecular CR. The patient then received the second cycle of consolidation chemotherapy with cytarabine (100 mg/m^2^ × 5 days) and daunorubicin (50 mg/m^2^ × 3 days), followed by a third cycle with cytarabine (70 mg/m^2^ × 5 days) and idarubicin (12 mg/m^2^ × 3 days).

As the impact of DMs on APL was uncertain, she was classified as having standard-risk APL. Thus, she was followed up without maintenance therapy from July 2022 with minimal residual disease (MRD) monitoring by *PML::RARα* mutation in peripheral blood and was confirmed MRD-negative. After 15 months, the patient’s blood examination showed pancytopenia, and she was diagnosed with relapsed APL with DMs based on her BM examination results. She was treated with reinduction therapy, a first and second cycle of consolidation therapy using arsenic trioxide (0.15 mg/kg/day). After a second cycle of consolidation therapy, BM examination revealed molecular CR. High-dose chemotherapy, followed by autologous stem cell transplantation (auto-SCT), was planned as a consolidation therapy. The patient received high-dose cytarabine (4 g/m^2^ × 4 days), followed by peripheral blood stem cell harvesting. She then received conditioning with busulfan (3.2 mg/kg/day) on days 1–3 and melphalan (70 mg/m^2^) on days 4 and 5, followed by the infusion of 4.4 × 10^6^/kg of CD34-positive cells. BM examination after auto-SCT showed molecular CR, and the patient was followed up. However, at 2 months after auto-SCT, real-time PCR with peripheral blood showed that the abundance of *PML::RARA* mRNA was 63 copies/μg RNA. The patient was determined to be positive for MRD. She provided her informed consent for differentiation therapy as a salvage therapy. She was started on oral ATRA and was MRD-negative with peripheral blood after 1 month. Fifteen months later, the patient is still receiving ATRA treatment and remains MRD-negative, with monthly monitoring.

## Discussion

Patients with AML harboring DMs generally have a poor prognosis due to resistance to chemotherapy. Amplification of oncogenes such as *MYC* or *MLL*, frequently observed in patients with AML and DMs [10, 11], has been implicated in resistance to chemotherapy. Moreover, AML with DMs is often associated with a monosomal or complex karyotype and *TP53* mutation, which are related to poor AML prognosis. This association is a possible reason for the poor prognosis of patients with AML and DMs [12]. To our knowledge, although several studies have reported APL-like cases lacking the translocation t(15;17) and PML::RARα mutation, Zuberi et al.’s case report remains the only report of APL with DMs [13–16] (Table 1). Therefore, the clinical significance of DMs in chemotherapy in patients with APL is unknown. The patient in our case is considered to have a poor prognosis because of the clinical course, including hematologic relapse and subsequent molecular relapse following auto-SCT. However, the patient did not exhibit *MYC* or *MLL* gene amplification and monosomal or complex karyotype [10–12].

**Table 1:** Literature review of the APL or APL-like leukemia with double minute chromosomes.

No.	Age	Sex	Karyotype	*PML:RARA*	References
1	78	M	46,XY,del(8)(q24.2),2-20dmin [10]/46,XY[20]	(−)	Frater et al., 2006
2	59	F	42–45,XX,-5,+6,-8,add(9)(p?),del(10)(q24),der(16)t(8;16)(q22;q24),del(18)(p11),	(−)	Mohamed et al., 1993
			der(20)t(17;20)(q21;q11),-22,+mar,10-100dmin		
3	76	F	45,XX,der(5)t(5;17)(q?;q12),del(8)(q22q24),-17,5-32dmin[31]	(−)	Brunel et al., 1995
4	67	F	44,X,-X,add(7)(q32),-9,der(12)t(12;17)(p13;q11.2)dup(17)(q21),-17,-21,+mar1,	(−)	Grimaldi et al., 2007
			+mar2,+dmin [9]/46,XX[10]		
5	87	F	43,X,-X,-9,del(9)(?p21),der(15;17)(p10;q10),-17,2-60dmin[21]	(−)	Kamath et al., 2008
6	61	M	46,XY,del(9)(p21),4-30dmin	(−)	Bruye’re et al., 2010
7	76	M	46,XY,7-50dmin[20]	(−)	Poddighe et al., 2014
8	71	M	44,X,-Y,del(5)(q13q33),-9,del(17)(p11.2),14-52dmin[15]	(−)	Sathyanarayana et al., 2025
9	57	F	46,XX,del(8)(q24.4),?del(14)(q11.22q11.22),t(15;17)(q22;q21) [4]/46,idem,2-20dmin[16]		Zuberi et al., 2010
10	44	F	46,XX,del(9)(q22q34),t(15:17)(q24;q21),1-21dmin[13]	(+)	Present case

In contrast, the patient described by Zuberi et al. [13] harbored a complex karyotype of APL and DMs with *MYC* gene amplification, yet remained in remission for 15 months, with ATRA. This may be because their case differed from ours regarding the use of maintenance therapy with ATRA, which has been reported to significantly prolong progression-free survival in high-risk patients with APL defined by WBC count at diagnosis [17]. Here, poor prognostic factors, including a WBC count > 10 000/μL at diagnosis, CD56 expression, complex chromosomal karyotype, FLT3-ITD mutation, and two or more gene mutations, were absent [4]. Additionally, our patient exhibited del [9](q22q34) as an additional chromosomal abnormality besides t(15;17). A previous study demonstrated that 9q deletion in APL might adversely affect prognosis; nonetheless, its clinical significance remains unestablished because of the limited number of cases. Therefore, 9q deletion in APL is not currently recognized as a poor-prognostic cytogenetic abnormality [4, 18]. Thus, we classified the patient as having standard-risk APL and followed her without maintenance therapy. DMs may also be associated with aggressive disease and poor prognosis in APL and AML, and the lack of ATRA maintenance therapy may have contributed to her first relapse. Although peripheral-blood MRD monitoring has been reported as beneficial for predicting relapse [19], she experienced hematologic relapse despite its application, potentially reflecting the clinical course of APL with DMs; more frequent monitoring and/or bone marrow MRD assessment may be required for patients with APL and DMs. The clinical course in the previous study was unclear because of discontinuation of follow-up at the patient’s request; thus, this is the first report that ATRA may be effective in preventing relapse in APL with DMs, even after a relapse. These findings suggest that patients with APL with DMs may represent a high-risk group predisposed to relapse but sensitive to differentiation therapy, offering long-term survival for patients with APL harboring DMs.

## References

[1] Guillerman RP, Voss SD, Parker BR. Leukemia and lymphoma. Radiol Clin North Am 2011;49:767–97. 10.1016/j.rcl.2011.05.004 21807173

[2] Al-Ibraheem A, Allouzi S, Abdlkadir AS. et al. PET/CT in leukemia: utility and future directions. Nucl Med Commun 2024;45:550–63. 10.1097/MNM.0000000000001846 38646840

[3] Burnett AK, Russell NH, Hills RK. et al. Arsenic trioxide and all-trans retinoic acid treatment for acute promyelocytic leukaemia in all risk groups (AML17): results of a randomised, controlled, phase 3 trial. Lancet Oncol 2015;16:1295–305. 10.1016/S1470-2045(15)00193-X 26384238

[4] Yokoyama Y . Risk factors and remaining challenges in the treatment of acute promyelocytic leukemia. Int J Hematol 2024;120:548–55. 10.1007/s12185-023-03696-7 38386203

[5] Yanada M, Tsuzuki M, Fujita H. et al. Phase 2 study of arsenic trioxide followed by autologous hematopoietic cell transplantation for relapsed acute promyelocytic leukemia. Blood 2013;121:3095–102. 10.1182/blood-2012-11-466862 23412094

[6] Sanz MA, Lo Coco F, Martín G. et al. Definition of relapse risk and role of nonanthracycline drugs for consolidation in patients with acute promyelocytic leukemia: a joint study of the PETHEMA and GIMEMA cooperative groups. Blood 2000;96:1247–53. 10942364

[7] Takeshita A, Asou N, Atsuta Y. et al. Impact of CD56 continuously recognizable as prognostic value of acute promyelocytic leukemia: results of multivariate analyses in the Japan adult Leukemia study group (JALSG)-APL204 study and a review of the literature. Cancers 2020;12:1444. 10.3390/cancers12061444 32492981 PMC7352829

[8] Iaccarino L, Ottone T, Alfonso V. et al. Mutational landscape of patients with acute promyelocytic leukemia at diagnosis and relapse. Am J Hematol 2019;94:1091–7. 10.1002/ajh.25573 31292998

[9] De Botton S, Chevret S, Sanz M. et al. Additional chromosomal abnormalities in patients with acute promyelocytic leukaemia (APL) do not confer poor prognosis: results of APL 93 trial. Br J Haematol 2000;111:801–6. 10.1046/j.1365-2141.2000.02442.x 11122141

[10] Huh YO, Tang G, Talwalkar SS. et al. Double minute chromosomes in acute myeloid leukemia, myelodysplastic syndromes, and chronic myelomonocytic leukemia are associated with micronuclei, MYC or MLL amplification, and complex karyotype. Cancer Genet 2016;209:313–20. 10.1016/j.cancergen.2016.05.072 27318442

[11] Thomas L, Stamberg J, Gojo I. et al. Double minute chromosomes in monoblastic (M5) and myeloblastic (M2) acute myeloid leukemia: two case reports and a review of literature. Am J Hematol 2004;77:55–61. 10.1002/ajh.20151 15307107

[12] Wang N, Yuan L, Jing Y. et al. Double minute chromosomes in acute myeloid leukemia and myelodysplastic syndromes are associated with complex karyotype, monosomal karyotype, TP53 deletion, and TP53 mutations. Leuk Lymphoma 2021;62:2466–74. 10.1080/10428194.2021.1919663 33904352

[13] Zuberi L, Adeyinka A, Kuriakose P. Rapid response to induction in a case of acute promyelocytic leukemia with MYC amplification on double minutes at diagnosis. Cancer Genet Cytogenet 2010;198:170–2. 10.1016/j.cancergencyto.2009.12.011 20362234

[14] Bruyère H, Sutherland H, Chipperfield K. et al. Concomitant and successive amplifications of MYC in APL-like leukemia. Cancer Genet Cytogenet 2010;197:75–80. 10.1016/j.cancergencyto.2009.11.001 20113841

[15] Poddighe PJ, Wessels H, Merle P. et al. Genomic amplification of MYC as double minutes in a patient with APL-like leukemia. Mol Cytogenet 2014;7:67. 10.1186/s13039-014-0067-6 25392715 PMC4228273

[16] Sathyanarayana SH, Bickford MA, Smuliac NA. et al. Complex genetic structural aberrations revealed by optical genome mapping in a case of APL-like morphology. Cancer Genet 2025;292-293:111–5. 10.1016/j.cancergen.2025.02.005 39999580

[17] Adès L, Guerci A, Raffoux E. et al. Very long-term outcome of acute promyelocytic leukemia after treatment with all-trans retinoic acid and chemotherapy: the European APL Group experience. Blood 2010;115:1690–6. 10.1182/blood-2009-07-233387 20018913

[18] Yamamoto K, Hamaguchi H, Kobayashi M. et al. Terminal deletion of the long arm of chromosome 9 in acute promyelocytic leukemia with a cryptic PML/RAR alpha rearrangement. Cancer Genet Cytogenet 1999;113:120–5. 10.1016/s0165-4608(99)00015-1 10484977

[19] Chendamarai E, Balasubramanian P, George B. et al. Role of minimal residual disease monitoring in acute promyelocytic leukemia treated with arsenic trioxide in frontline therapy. Blood 2012;119:3413–9. 10.1182/blood-2011-11-393264 22374701

